# Supplementary material for: Prevalence and determinants of prehypertension (elevated blood pressure or high normal BP) according to different classifications in India during 2015–2021: Evidence from the large national surveys
Source: PLoS One. 2025 Jun 11;20(6):e0325437. doi: 10.1371/journal.pone.0325437 (PMC12157057; doi:10.1371/journal.pone.0325437)
Supplement: S1 Table — (DOCX) [file pone.0325437.s002.docx]

**Supplementary Appendix – 1**

**S1 Table. Blood pressure classifications according to AHA/ACC, JNC7/8 and IGH-IV /ESC guidelines**

| **S.no.** | **Classification** | **Category** | **Systolic BP** | **Diastolic BP** |
| --- | --- | --- | --- | --- |
|  | **AHA/ACC** | Normal | <120 | <80 |
|  |  | Elevated BP | 120-129 (and) | <80 |
|  |  | High BP HTN – STAGE 1 | 130-139 (or) | 80-90 |
|  |  | High BP HTN – STAGE 2 | >=140 (or) | >=90 |
|  |  | Hypertensive crisis | >180 (and/or) | >120 |
|  |  |  |  |  |
|  | **JNC 7/8** | Normal | <120 | <80 |
|  |  | Pre hypertension | 120-139 | 80-89 |
|  |  | Hypertension– STAGE 1 | 140-159 | 90-99 |
|  |  | Hypertension – STAGE 2 | >= 160 | >=100 |
|  |  |  |  |  |
| **3.** | **IGH- IV /ESC** | Normal | <130 | <85 |
|  |  | High- Normal BP | 130-139 (or) | 85-89 |
|  |  | Hypertension– STAGE 1 | 140-159 | 90-99 |
|  |  | Hypertension – STAGE 2 | 160-179 | 100-109 |
|  |  | Hypertension STAGE 3 | >= 180 | >= 110 |
