# Supplementary material for: Prevalence and determinants of prehypertension (elevated blood pressure or high normal BP) according to different classifications in India during 2015–2021: Evidence from the large national surveys
Source: PLoS One. 2025 Jun 11;20(6):e0325437. doi: 10.1371/journal.pone.0325437 (PMC12157057; doi:10.1371/journal.pone.0325437)
Supplement: S1 Text — (DOCX) [file pone.0325437.s003.docx]

**Supplementary Appendix- 2**

**S 1 Text. Importance of LISA significance and Moran’s index**

Local Indicators of Spatial Autocorrelation (LISA) and Moran’s Index are two measures of spatial correlation.

**Moran’s Index** is a correlation of value between actual value of a place with the average of its neighbor area. Positive and significant Moran’s I value depicts clustering of like values and these values can be either high or low or even combination of both. The negative and significant Moran’s I value depicts presence of spatial outliers. Moran’s I is a single value for the whole spatial pattern and does not provide the location of clusters. [1]

**Local Indicators of Spatial Autocorrelation (LISA)** displays the relationships between each observation and its surroundings, instead of providing a single summary of relationships across the map. It indicates significant spatial autocorrelation for each location. A LISA has two important principles; Firstly, it provides a statistic for each location with an assessment of significance. Secondly, it helps to establish a proportional relationship between the sum of the local statistics and global indicator of spatial association.

This entity does not serve as a single summary statistic but a score, which elaborates on the spatial structure in the data. The significant value of LISA helps to identify the set of contiguous location, which are labeled as “local spatial clusters” or “Hot spots”. These clusters can be correlated based on high values or low values and combination of both. It also displays the spatial outliers.

The color-coded areas show significant correlation local statistic whereas the undefined or uncolored areas depict that there is no significant spatial correlation. The high- high and low- low are positive spatial autocorrelation whereas high- low and low- high are negative spatial autocorrelation. [1]

References

[1.] Nilima

, Asha Kamath, Karthik Shetty. Prevalence, patterns, and predictors of diarrhea: a spatial-temporal comprehensive evaluation in India. BMC Public Health (2018) 18:1288 . <https://doi.org/10.1186/s12889-018-6213-z>
