# Supplementary material for: Prevalence and determinants of prehypertension (elevated blood pressure or high normal BP) according to different classifications in India during 2015–2021: Evidence from the large national surveys
Source: PLoS One. 2025 Jun 11;20(6):e0325437. doi: 10.1371/journal.pone.0325437 (PMC12157057; doi:10.1371/journal.pone.0325437)
Supplement: Attachment — Submitted filename: Response to Reviewers .docx [file pone.0325437.s005.docx]

**1Additional Editor Comments:**

The critical comments mainly related to analytical approach are noted below.

**Dear Editor and Reviewers,**

Thank you very much for giving your precious time to expertly review and narrate detailed constructive comments on our manuscript. It has added great improvements and insights to our manuscript therefore, upgraded it significantly.

We have incorporated the modifications and additions as suggested by the learned editors and reviewers. The raised comments have been addressed as follows:

| 1. | Study lacks clarity in methods and analytical framework. For instance, Table 2 is confusing because it state ‘Prevalence’ and it shows Mean Systolic BP and Mean Diastolic. It difficult to ascertain what authors wanted to convey based on this very table and showing from NFHS4 to NFHS 5 there is an increase in Hypertension across all classification and participant characteristics. From the analytical point of view, it many not be a right approach to check the agreement between different classifications based on the these findings. | Thank you for highlighting this concern.  As the Table 2 title reads the prevalence of prehypertension across different classifications except for the Mean SBP, Mean DBP and Mean age, all the values in table depicts only prevalence across different classifications. Hence, for easy understanding and not to confuse the readers as suggested by the editor and reviewer- 1, we have revised the Table 2 and removed the Mean SBP, Mean DBP and Mean age data from the table and included that in the text of results section.  Now the revised Table 2 shows only the prevalence of prehypertension across different classifications and various variables. |
| --- | --- | --- |
| **2.** | Also, authors have stated that they dropped missing cases in the footnote of Table 1. This is a wrong approach without discussing/describing the nature and extent of missing cases in the data. There is various proven data imputation ways which could be used for missing data. | Thank you for the comment. You have rightly pointed out the concern about missing cases. Our main concern was about different classifications of prehypertension prevalence at national and sub-national level. Imputation of missing cases would be another concern but that is not part of our objectives. With the help of literature supports, we have dropped the missing cases. |
| **3.** | The NFHS fifth round survey provides information for 724,115 women (age 15-49 years), and 101,839 men (age 15-54 years). In such a skewed distribution of samples between men and women, what additional analytical strategies have been incorporated, is not reflected? Even the age distribution between men and women are not similar and how this could affect the overall prevalence of hypertension between men and women needs to be examined. | Thank you for the comment.  We have not done the unit level analysis, as all the estimates are computed for the household level.  The age groups (age 15-49 years) for females and (age 15-54 years) for males in the text were mentioned by mistake, and we have deleted the same.  The distribution for both gender (Male and female) according to age groups are similar in NFHS-4 and NFHS-5 as shown in Table 1.  [Line 147,148] |

Reviewer's Responses to Questions

**Comments to the Author**

1. Is the manuscript technically sound, and do the data support the conclusions?

The manuscript must describe a technically sound piece of scientific research with data that supports the conclusions. Experiments must have been conducted rigorously, with appropriate controls, replication, and sample sizes. The conclusions must be drawn appropriately based on the data presented.

Reviewer #1: Yes

Reviewer #2: Partly

Reviewer #3: Partly

2. Has the statistical analysis been performed appropriately and rigorously?

Reviewer #1: Yes

Reviewer #2: Yes

Reviewer #3: Yes

3. Have the authors made all data underlying the findings in their manuscript fully available?

The [PLOS Data policy](http://www.plosone.org/static/policies.action#sharing) requires authors to make all data underlying the findings described in their manuscript fully available without restriction, with rare exception (please refer to the Data Availability Statement in the manuscript PDF file). The data should be provided as part of the manuscript or its supporting information, or deposited to a public repository. For example, in addition to summary statistics, the data points behind means, medians and variance measures should be available. If there are restrictions on publicly sharing data—e.g. participant privacy or use of data from a third party—those must be specified.

Reviewer #1: Yes

Reviewer #2: Yes

Reviewer #3: Yes

4. Is the manuscript presented in an intelligible fashion and written in standard English?

PLOS ONE does not copyedit accepted manuscripts, so the language in submitted articles must be clear, correct, and unambiguous. Any typographical or grammatical errors should be corrected at revision, so please note any specific errors here.

Reviewer #1: Yes

Reviewer #2: Yes

Reviewer #3: No

Reviewer #1: The authors have highlighted an important issue which has huge ramifications on policy issues. Various definitions of high normal, elevated and pre hypertension add confusion to the end users and need to be streamlined. Hence, a uniform cut off must be defined. It is relevant to point out that the authors have displayed the district level prevalence of pre-hypertension, wherein, ArcGIS was used to create maps to visually depict the differences when comparing the NFHS-4 to NFHS-5. GeoDa was also used to compute local indicators of spatial association which is a novel way to present data.

Below mentioned Minor corrections must be addressed

| 1. | In the abstract, results section (line 44 and 45) should read as “The prevalence of prehypertension as per JNC7/8 guidelines (35.8% vs 48.8%), 2017 ACC/AHA (6.1% vs 8.8%) and IGH-IV (12.5% vs 20.8%) was higher in NFHS-5 when compared to NFHS-4 instead of as compared to NFHS-4. | Thank you for the input, we have made the corrections in line 45 and 46 in revised manuscript . |
| --- | --- | --- |
| 2. | Line 89….begin with In a cohort study from India, it was reported ….instead of A cohort study from India, it was reported….. | Thank you for the suggestion, the edit has been done in line 91, 92. |
| 3. | In lines 95, 96, 97, 98…. Combine these two sentences into a single sentence instead of two separate sentences- “The American Joint National Committee (JNC) identified prehypertension as separate category in its seventh recommendation and defined it as having systolic blood pressure (SBP) of 120-139 mm Hg or diastolic blood pressure (DBP) of 80-89 mm Hg [6]. Though, it was not addressed in the Eighth Joint National Committee (JNC 8, 2014) report [13]”. | Thank you for the suggestion. The sentence has been combined and edited as per suggestion [line 98,99,100]. |
| 4. | Although not essential, it will be useful for the readers to appreciate the definitions by JNC, ACC, ESC very carefully as readers often get confused by the and /or categories. The authors are advised to highlight the key definitions in a supplementary table OR the authors are advised to keep the definitions in bold as follows: ACC definition - Elevated Blood Pressure’ when an individual has SBP of 120-129 mm Hg and DBP <80 mmHg JNC 7 definition of prehypertension - defined it as having systolic blood pressure (SBP) of 120-139 mm Hg or diastolic blood pressure (DBP) of 80-89 mm Hg European Society of Cardiology (ESC) and European Society of Hypertension (ESH) define “High Normal blood pressure” if SBP is 130-139 or DBP is 85-89 mm Hg | Thank you for the important note.  A supplementary table (S1) has been added comprising of all the classifications used in this paper for better clarification and understanding. |
| 5. | Line 105, 106 – please modify. Although the IGH IV have used the term high normal found in ESC, it is not the same. High normal definition by IGH IV – please double check and define it in the document. It is as follows IGH IV defines high normal BP: systolic BP of 130-139 mm Hg and diastolic of 80-89 mmHg | IGH-IV follows ESC guidelines.  We have checked and the range for **DBP is 85-89 mmHg.** |
| 6. | Lines 113 and 114 read as “In this context, present study was conducted to estimate burden of prehypertension (elevated blood pressure or high normal BP) and its determinants based…..” Does this mean that they have considered both elevated blood pressure and high normal BP under prehypertension category? Clarification on this is required. | Thank you for the query, we want to clarify that different classifications have used different terminologies and cut offs for prehypertension. It is described in text and again here for clarification.  **JNC- 7/8** used the term **prehypertension**  **ACC/AHA** used the term **elevated BP**  **ESC/IGH-IV/ISH** have used the term **High normal BP** |
| 7. | Line 137 - Although it is commonly understood that “Study population, design, sample size and sampling technique were similar for NFHS-4 and NFHS-5”, references provided for the same (at least on sample size, sampling techniques) will aid the readers (belonging to non-Indian countries and similar LMICs settings) to objectively verify the same. The authors must be commended for mentioning the study population and procedures in detail – but they have failed to provide references that substantiate their sentences under study population and procedures. References 21, 22 and 23 that the authors have provided do not allow the reader to verify the sample size and sampling technique used for both NFHS 4 and NFHS 5. | Thank you for your appreciation.  The readers can further read sample size and sampling technique in NFHS 4 and 5 by exploring the references 21 and 22  References 21 and 22 cited in the methodology provide detailed information regarding the sampling techniques, study population and sample size. |
| 8. | Line 143 mentions NFHS 5 “recruiting 724,115 women (15-49 years), and 101,839 men (15-54 years).” Were similar age group categories of 15-49 years for females and 15-54 years for males also captured in NFHS 4? | Thank you for the query about age group distribution.  The age groups (age 15-49 years) for females and (age 15-54 years) for males in the text were mentioned by mistake, and we have deleted the same.  [Line 147 and 148]  The distribution for both gender (Male and female) according to age groups are similar in NFHS-4 and NFHS-5 as shown in Table 1. |
| 9. | Although the authors have mentioned that same instrument was used for measuring BP (HEM 8712) and procedures during NFHS 4 and NFHS 5 were the same (reference 21 and 23, which the authors have cited - do not mention this), it is highly susceptible to inter person variations as the people who measured the BP in 2015 (NFHS 4) would not have been the same in NFHS 5 (2019). Were they trained and was inter person variability measured? If not, this must be mentioned in the study limitations. | Thank you for pointing this out.  The field staff involved in survey was adequately trained and the NFHS-4 and NFHS-5 surveys are cross sectional surveys conducted over different period of time on different set of population. |
| 10. | Line 168 does not read correctly. “In these definitions, for calculations of BP, he/she reported not using antihypertensive medicines currently”. Suggested to write in clear terms. Did the authors meant to convey – For the study purpose and for determining the prehypertension/high normal/elevated BP, we ascertained that the participants were not using antihypertensive medications currently? If so, please state it explicitly | Thank you for the suggestion,  We have reframed the sentence for better comprehension. [Line 176-178] |
| 11. | Lines 172, 174 and 174 - The independent variables considered after literature review for evaluation-included age groups, sex, education status, social groups, residence, wealth index, tobacco consumption, alcohol consumption and National Rural Health Mission (NRHM) state categorization. Why only these variables? Reasoning must be included. What literature review was perfomed? | This is the secondary analysis highlighting the prevalence of prehypertension across different classifications, after literature review it was concluded that socio demographic variables were included to highlight the population characteristics.  **Q: Why only these variables were used?**  **A**: As the objective of the study was to explore the trend in burden and its determinants and not to find the associated risk factors in detail.  After detailed literature review, the independent variables included in the analysis for this study were significant.  Kindly refer the following references : [24,25,26,27,28,29,30]  [Line 181- 184] |
| 12. | Line 179 mentions rural and urban stratification for residence. Would it be possible to present data as rural, urban, tribal and peri urban for residence variable? | Data cannot be divided into tribal and peri urban as NFHS METHODOLOGY presents data in two categories only (rural and urban).  Data were available for all the states in categories of rural and urban areas.  Tribal population is a subgroup population which can be analyzed separately. |
| 13. | Line 177 - Educated till primary needs to be defined as most readers would want to understand what primary and secondary education mean? | Thank you for the query.  We have mentioned in the article where primary education consists of up to 5^th^ class and secondary education refers to class 6^th^ -12^th^. [Line 187] |
| 14. | Line 190 – “We defined prehypertension and its equivalent terms as per JNC7/8, 2017 ACC/AHA, and IGH-IV.” In the introduction, the authors themselves have showcased the different definitions and the overlap. It is not cleat what cut offs they have used for defining pre hypertension here. They must mention. | The same cut off for prehypertension and equivalent terms as per different classifications have been used as previously stated in introduction and outcome variable sections.  The results have been formulated using and comparing all three classifications.  It’s mentioned in Supplementary table 1 depicting all the cut offs for all classifications, thereby, providing further detailed reading. |
| 15. | Line 203 – “We undertook the weighted analysis to present the descriptive statistics such as mean SBP, DBP and age. A clear description of weighted analysis must be presented. What weights have they used and what was the procedure? Detailed description must be provided. | We have computed all the estimates by using national sample weight which is already given in the dataset. The NFHS computed the national weight using population proportion as per census of India. |
| 16. | A supplementary appendix could be appended to describe the importance of LISA significance and Moran’s index so that readers understand why they have used these techniques. | Thank you for highlighting this important aspect. We have added supplementary document (S2) elaborating the concepts of LISA significance and Moran’s Index. |
| 17. | Lines 230 and 231 -Sentences need to be clearly spelt. “and meager 1.1% was above of 50 years and above in NFHS-4 while in NFHS-5 about 30% was in 50 years and above category.” Better way to state would be – Only 1.1% was above 50 years category in NFHS 4 , while it was 30% in NFHS 5. | We have reframed the sentence as suggested.  [Line 240,241] |
| 18. | Use comma separators for all numbers in table 1 since the data is in lacs. Example: 644348 should read as 6,44,348 and so on… | Thank you for pointing this out, the correction has been done. |
| 19. | Table 1 for wealth index – authors should define in subscript under the table…poor, poorest, richer and richest | Thank you for this valuable suggestion.  The description of wealth index categorization is very detailed and cannot be accommodated under the subscript in table. Hence, we have added good references [31,32] for details regarding the wealth index calculation and quintiles.  This should help the readers in understanding the wealth index used in our analysis.  [Line 190,191] |
| 20. | Line 252 – “132 (IGH)”. Mention IGH -IV | Thank you, the correction has been done |
| 21. | Line 264. Bracket should be closed after 10.4% | The correction has been done |
| 22. | Line 265 should read as “Figure 1 depicts the ….” | The correction has been done |
| 23. | Line 253 – should read as “Mean age for prehypertension was” and not is…. | Thank you, the correction has been done |
| 24. | Line 255 - Rewrite this sentence “The prevalence of prehypertension (and related terms) was higher with increasing age in JNC 7/8 and IGH-IV but this trend is not present as per ACC/AHA though prevalence was highest for age group 50 years and above.” Write in clear terms. | Thank you for the suggestion, the sentence has been reframed and is now more comprehensible. |
| 25. | Line 276 - Depict estimate and 95% CI clearly. “[Odds ratio (OR)= 0.64,0.64,0.55]”. This should read as OR = 0.64 (95% CI: 0.55 – 0.64) | Thank you for the concern, but the figure represented in text corresponds to Odd’s ratio for the category as per the three classifications and not the confidence interval limits. |
| 26. | Similarly do it for line 277 and in all subsequent lines where you showcase the estimate and 95% CI. | As mentioned above, the figures are Odd’s ratio itself for the three classifications and not confidence interval limits. |
| 27. | Line 288 - This sentence needs to be rewritten clearly. “Belonging to ‘low-focused’ states from being a risk factor across classifications in NFHS- 4[Odds Ratio (OR)=1.19,1.26,1.14], to being protective in NFHS-5 [Odds Ratio (OR) =0.76,0.84,0.84].” | Thank you for the input, the sentence has been reframed.  [Line – 296,297,298] |
| 28. | Table 2 . This is my main concern. Title cannot be prevalence as the authors are only showing mean values of SBP and mean values of DBP and as they have mentioned mean age under the age category. Is it the prevalence in that age group or is it the mean age in that age category? Upon checking the difference between NFHS 5 and NFHS 4 – say for example for overall the value 42.2 – 32.6 = 9.6 suggest that they mean prevalence. In that case, mean age under the heading age in years is misleading. Subtitle each category as prevalence in different age categories, prevalence based on gender, prev based on social groups, prev based on residence, prev based on wealth quartiles etc. Table 2 needs major revision. | Thank you for the kind concern, we have calculated the prevalence of SBP and DBP across all the variables and the mean data. The value in tables depicts prevalence only except for the row where the mean is mentioned. **Henceforth, to avoid any confusion we have removed the data for mean SBP, mean DBP and Mean age data from the table and mentioned in the results text.**  **It is the prevalence in the different age groups.**  **The revision in Table 2 is done and all the value snow in table depicts the prevalence.** |
| 29. | Table 3 - Any reason as to why the estimate for OR and the 95% CI is consistently less than 1 as the age advances in the ACC category while it is more than 1 in JNC and IGH IV both in NFHS 4 and 5? | Thanks again for this comment. If we see the classification, the cut off for pre-hypertension as per JNC and IGH IV is more or less same where ACC follows the very different cut off for the classification for the same. As age advances, the risk of pre-hypertension is increasing. Further, as per ACC criteria, may be people are more shifting from pre-hypertension to hypertension that’s why lesser odds found among higher age groups. |
| 30. | Table 3- change the subheading to read as OR (95% CI) and not CI at 95% | Thank you for the suggestion, the correction has been done |
| 31. | Line 332 – it is not clear what the authors suggest by positive autocorrelation | Thank you for the comment, we have added supplementary document (S2 Text) explaining the concept of both positive and negative auto correlation. [Line 344] |
| 32. | Line 333 and 334 – “prevalence of prehypertension among districts, a total of 244 districts in NFHS-4 and 242 districts in NFHS-5 had significant neighborhood association.” This should read as prevalence of prehypertension among districts. A total of 244 districts in NFHS-4 and 242 districts in NFHS-5 had significant neighborhood association. | Thank you, the sentence has been edited.  [Line 341,342] |
| 33. | It will benefit the readers if the concept and implications of Moran’s index is explained briefly in one or two sentences. | Thank you for the suggestion, we have added Supplementary document (S2 Text) which explains the concept of Moran’s index. |
| 34. | Line 369-371- This is the main finding of the paper. “Our key finding shows that nearly 50% of 369 the Indian population aged ≥ 15 years has prehypertension using the most popular JNC7/8 criteria. There is striking difference in this estimate at 8.8% and 20.8% using ACC/AHA (2017) and 2019 IGH-IV criteria respectively.” Please state this explicitly that this is based on NFHS 5 data. | Thank you for pointing it out, we have mentioned explicitly that this finding is based on NFHS-5 data. [Line 374-376] |
| 35. | Line 386 and 387 – “Burden calculated by IGH is less than half of burden by JNC guidelines (50% vs 387 20.8%).” State this clearly as ‘prevalence burden for prehypertension based on definition by IGH IV…. | Thank you for the suggestion, the correction has been done.  [Line 391-393] |
| 36. | Line 391 – add the word ….”pressure to label different categories of hypertension which increase the likelihood of poor health outcomes” | Thank you, the sentence has been edited  [Line 397, 398] |
| 37. | Line 397 – add the word “Analysis of prehypertension from NFHS-4 data showed 36% of participants had prehypertension as per JNC (42.5 % in…..” | Thankyou the correction has been done.  [Line 403-405] |
| 38. | Check references for line 403…”than that of individuals with normal BP [12,4-47].” | Thank you for the input, we have corrected the reference.  [Line 409] |
| 39. | Line 412 – “A South American country (Peru, 2017-2018) reported 21.2% burden.” Burden of what? | Thank you for pointing it out, we have added that it’s the burden of prehypertension.  [Line 417,418] |
| 40. | Line 419- 421- This sentence is confusing. Please reword it appropriately….” Our analysis found mean increase in population-level systolic as well as diastolic BP from NFHS-4 to NFHS-5 and more than 120 mm Hg for systolic range while > 80 mm Hg was DBP except for AHA/ACC.” | Thank you for the suggestion, the correction has been made and the sentence has been reframed.  [Line 424- 427] |
| 41. | Line 422- “or stagnating trends in LMICs”…of what? | Thank you for pointing it out, we have added the missing part of the sentence which was “trends in LMICs of blood pressure”.  [Line 428,429] |
| 42. | Line 457 to 459- Please paraphrase or modify this sentence as this appears to be taken verbatim from the reference. “There are basic sex-related differences, which contribute to differences in controlling blood pressure (i.e. renin—angiotensin system and sympathetic nervous system). Denton et al, described sexual dimorphism and the influence of sex steroids on genetic, hormonal and biochemical pathways for functioning of cardiovascular system in men and women [73].” | Thank you for the suggestion, we have paraphrased the write up.  [Line 461- 467] |
| 43. | Line 472 – please give evidence for this statement “Education had a protective effect across classifications as evident form NFHS 5 data, which was variable in previous survey” | Thank you, in order to clarify, this statement is inference of present study results.  Similar evidence has been found in other studies as cited in the text with reference nos. [28,52,53,67].  [Line 477-479] |
| 44. | Line 541 – read as measured | The correction has been done.  [Line 547] |
| 45. | Check grammar and English language usage for lines 547 to 549 in conclusion | Thank you for the suggestion, the sentence has been edited.  [Line 554- 558] |
| 46. | Link for data sets Link in the manuscript on page number 4 is nonfunctional. Correct link is: https://dhsprogram.com/data/available-datasets.cfm The authors have added an extra space between dhsprogram and .com – hence the link is non functional. | Thank you for pointing out, the functional link for data sets has been added. |

Reviewer -2

General Comments

| S.No | Comments By Reviewer | Reply |
| --- | --- | --- |
| 1. | The title is too long | Thank you for your kind suggestion.  The elevated BP and High normal BP are equivalent term for prehypertension which are used in different classifications, and this has been discussed in detail in introduction.  So, we want to draw the attention of readers to this important issue of different classifications with different cut offs and different terminologies that add to the confusion in defining a singular criterion for prehypertension. |
| 2. | The background section of the abstract section begins with the aim of the study, which needs correction. | Thank you for the highlighting this important point. We have reframed the background in abstract section. |
| 3. | Avoid use of abbreviations in the abstract section | Thank you for pointing it out. The correction has been made. |
| 4. | The language, spelling and grammar is needing meticulous correction. | Thank you for the feedback. We have done proof reading for spelling, language and grammar in between lines rigorously. All the corrections have been done. |
| 5. | Try to stick to the PLOS ONE Journal guideline. | Thank you, we have strictly complied with all the PLOS ONE journal guidelines. |
| 6. | The methods section needs brief explanation and should address data quality measures | It is narrated in methods section, further explained her. In order to establish the prevalence of hypertension, NFHS-4,5 tested the blood pressure of eligible respondents using OMRON blood pressure monitor. Each respondent had their blood pressure taken three times, with a five-minute interval each test. The despondent received the results on a health card. The health investigator has explained to the respondent the significance on the findings. In addition, the respondent also advised to visit healthcare if needed. In analytical part, we completely ignored the first reading because it invariably produced biased results (Arku et al., 2018). Additionally, NFHS-5 report also adopted the same procedure to estimate the pre-hypertension (IIPS and ICF, 2021) Therefore, to calculate the pre-hypertension, the average of the last two systolic and diastolic readings was employed.  Arku, R. E., Ezzati, M., Baumgartner, J., Fink, G., Zhou, B., Hystad, P., & Brauer, M. (2018). Elevated blood pressure and household solid fuel use in premenopausal women: Analysis of 12 Demographic and Health Surveys (DHS) from 10 countries. *Environmental research*, *160*, 499-505. |
| 7. | Why have you used various classifications? And which is mostly used in India? | Thank you for the query.  Different classifications and cut-offs determine the prevalence of prehypertension and, subsequently, policymaking for controlling and preventing hypertension, and formulating screening programs. Thus, it is critical that uniform and appropriate cut offs be determined for prehypertension.  In India, **JNC-8** is used at present. |
| 8. | The case to variable ratio is very small, according to the presentation in the result section | Thank you for the comment, but it is not the case.  Appropriate statistical analysis has been done and we have reported the results as found. |
| 9. | What was done for those with elevated Blood pressure? | It is a secondary analysis of population-based data from India conducted on data sets of NFHS-4 and NFHS-5.  The individuals with hypertension were encouraged to see the doctor for full evaluation as per the NFHS survey.  Please refer to the document (reference no 21, page 46).  No specific intervention for individuals with prehypertension was mentioned in either document. |
| 10. | The results need further logical flow, simplicity and clear description. | Thank you for the comment. We have meticulously reviewed and edited the results for logical flow, simplicity, and clear description. |
| 11. | Reference: Use up to date references. | Thank you, we have added new references in the article. |
| 12. | Statistics: Needs major revision E.g., you haven’t reported confidence interval while presenting percentages. Where is the mean/median age of elevated blood pressure? | We have reported the confidence interval for both percentage and mean age of elevated blood pressure in the revision.  The mean/median age of elevated blood pressure is mentioned in the results section. |

Reviewer #3: The manuscript requires professionally editing for English language as there are typos and grammatical errors.

Reviewer #3

| 1. | The Introduction needs to be drafted again highlighting the global indicators and then focusing on country statistics with current references. | Thank you for the comment, we have revised the introduction as per the suggestion and both recent global and country statistic have been added.  REF (11,12,19) are recent (2021,2020,2023) |
| --- | --- | --- |
| 2. | The Introduction should also provide a strong justification of conducting the study as similar studies have already been published. | Thank you,  Strong rationale for the study has been provided in the introduction section and highlighted the fact that it is an important public health concern which can have huge ramifications on population health outcome. In addition, to best of our knowledge no study is available comparing all classification with such large sample size.  Moreover, ArcGIS was used to create maps to visually depict the differences when comparing the NFHS-4 to NFHS-5 and GeoDa was also used to compute local indicators of spatial association which is a novel way to present data. |
| 3. | It should be highlighted what the study adds to the existing knowledge base and | Thank you for the suggestion.  It’s a unique large population-based analysis which has highlighted the comparison for all classifications of hypertension with different cut offs at national level.  Additionally, ArcGIS was used to create maps to visually depict the differences when comparing the NFHS-4 to NFHS-5 and GeoDa was also used to compute local indicators of spatial association which is a novel way to present data. |
| 4. | In discussion component the limitations should be clearly highlighted. | Thank you for the comment,  We have mentioned the limitations at the end of discussion in last paragraph.  [Line 544- 547] |
