# Supplementary material for: Prevalence and determinants of prehypertension (elevated blood pressure or high normal BP) according to different classifications in India during 2015–2021: Evidence from the large national surveys
Source: PLoS One. 2025 Jun 11;20(6):e0325437. doi: 10.1371/journal.pone.0325437 (PMC12157057; doi:10.1371/journal.pone.0325437)
Supplement: Attachment — Submitted filename: Response to Reviewers.docx [file pone.0325437.s006.docx]

**Dear Editor and Reviewers,**

**We would like to extend our sincerest gratitude for the invaluable feedback provided for the manuscript. Your constructive comments and insightful suggestions have been instrumental in improving our manuscript.**

| **S.No.** | **Reviewer’s Comment** | **Author’s Response** |
| --- | --- | --- |
| 1. | The title should also strictly address whether it is evaluation or research? Finally, the title is too long and it is better to shorten based on the criteria’s of the title. | Thank you for your kind suggestion.  It is research, as mentioned in the title “**Evidence from the large national surveys of India**”, and we have analyzed data from these cross-sectional surveys (National Family Health Survey) conducted between 2015 and 2021 to determine the prevalence and determinants of prehypertension among Indian population.  It is also noteworthy that details regarding the analysis of this population level data is mentioned in the methods section.  About the title we want to bring to your kind attention that, the **elevated BP and High normal BP are equivalent term for prehypertension** **which are used in different classifications,** and this has been discussed in detail in introduction and methodology as well.  So, we want to draw the attention of readers to this important issue of different classifications with different cut offs and different terminologies that add to the confusion in defining a singular criterion for prehypertension.  Therefore, **“Elevated blood pressure or high normal BP”** was added in title to help the readers in understanding the different terminologies used for prehypertension which probably is making the title long. |
| 2. | The manuscript is in general wide and should be revised accordingly. | Thank you,  As per your kind suggestion, we have revised the manuscript.  Taking into account the objectives of this study and we have analysed the National level data estimates from two rounds of National Family Health Survey (NFHS-4, NFHS-5) for prehypertension based on three different classifications in a comprehensive manner. The manuscript has been written in detail with explanations for different classifications and differences in prevalence estimates. |
| 3. | The abstract needs more clarity E.g., the type of study design is not described. | Thank you for the comment.  The method section of the abstract has been revised for better understanding.  We conducted secondary analysis on data from the National Family Health Surveys 4 and 5. The data sets of the NFHS are available at the site <https://dhsprogram.com/data/available-datasets.cfm> .  The data availability statement has also been added at the end of the manuscript. |
| 4. | The background is incomplete. E.g., The gap and its consequence are not well described, efforts are not mentioned. | Thank you for your important comment,  The introduction has been revised and, gap and consequences have been elaborated. Strong rationale for the study has been provided in the introduction and highlighted the fact that it is an important public health concern which can have huge impact on population health outcome. In addition, to best of our knowledge no study is available comparing all classification with such large sample size.  In addition, we have described in the introduction, the consequences for having different classifications for prehypertension. Moreover, ArcGIS was used to create maps to visually depict the differences when comparing the NFHS-4 to NFHS-5 and GeoDa was also used to compute local indicators of spatial association which is a novel way to present data. |
| 5. | The study design is lacking. | Thank you for the comment.  As previously addressed ,  We conducted secondary analysis on data from the National Family Health Surveys 4 and 5. The data sets of the NFHS are available at the site <https://dhsprogram.com/data/available-datasets.cfm> .  The data availability statement has also been added at the end of the manuscript. |
| 6. | The study setting lacks brief description | Thank you for the query,  The details regarding study setting have been mentioned in the methods section under Study population and procedures.  Please refer to **Line 155- 160 in the main article file.**    Please refer to **reference no. 21 and 22** for further reading. |
| 7. | Measurement and the procedures are not presented in detail | Thank you for the comment.  We have added the details regarding this in the methodology section as per your kind suggestion.  **Line 167- 172 in main article file.**  “The process and instrument for measuring blood pressure were the same for NFHS 4 and 5. BP reading was measured for women and men above 15 years of age in the selected households using a standard digital blood pressure device. BP was measured three times for each individual (using Omron™ HEM-8712), with at least five minutes gap between each measurement. The unit of BP measurement was mm of Hg [21,22].”  All the details regarding training, measurements, and procedures followed are described in detail in the NFHS 4 and 5 documents .  Please refer to **Reference no. 21 and 22 for further reading.** |
| 8. | The type of analysis and the reason behind your selection and its consistency throughout the document | Thank you for your kind suggestions.  Please refer to the sub section ‘**statistical Analysis’** mentioned under **‘methodology’** section.  In ‘statistics’ section, we have clearly mentioned all the statistical approach adopted in the study in detail  **Line No. 203- 232 in the main article file.** |
| 9. | Measurement E.g., the position of the study participant while taking Blood pressure and intake before the measurement | Thank you for the comment.  Please refer to **Line 167- 172 in the main article file.**  All the details regarding training, measurements, and procedures followed are described in detail in the NFHS 4 and 5 documents .  Please refer to Reference no. 21 and 22. |
| 10. | The randomness of the study participants is doubtable E.g., most of them (>50%) were those aged 30-39. | Thank you for the concern.  However, brief description has been mentioned in the manuscript about details of simple random method used for selection of participants in a two stage sampling approach.  **Line no. 161- 170 in the main article file.**  Please refer to Reference no. 21 and 22 further reading. |
| 11. | The age category is not equal | Thank you for the query,  To generate the table, we have categorized the age as per their homogenous lifestyle, namely, below age 30 years, middle age group, older adults, and ageing population.  In table, the age is categorized as, less than or equal to 30 and 31-40 which doesn’t overlap.  **Line 194-195 in the main article file.** |
| 12. | The result is not presented in logical, simplified and coherent way E.g. the table sequence, presentation of response rate in the methods section. | Thank you for your query.  We have taken your input and rearranged the sequence of our results. We aligned the text description in sequence with the tables and figures.  Moreover, we have edited certain sections to improve the readability.  **Line 294- 296, 300- 304, 323- 325 in the main article file.** |
| 13. | The discussion is not discussion. Try to refer on how to write strong discussion. | Thank you for the comment.  We have revised the discussion as per the reviewer’s suggestion, and summarised the key results with respect to study objectives.  We have written discussion on all the key findings of our study, compared results with published literature, and also added few new references. Limitations of our study have also been mentioned at the end of discussion. |
| 14. | Improper language and grammar need revisit E.g., the word ‘consensus’ is used at least four times in the discussion section. | Thank you for the comment.  Revisions for grammar and language have been done and repetition of words have been removed. |
| 15. | The recommendation should follow national initiatives of the country and needs to be pointing. | Thank you for the comment.  We have revised the recommendations section and included the inputs from National initiatives being implemented in India under the National Program- Non Communicable Diseases (NP-NCD) and added few more recommendations as per the suggestion.  **(Line 546- 552, 567- 569 in the main article file).**  For in depth details about the current measure please Refer to R**eference No.- 67** |
