# Supplementary material for: Prevalence and determinants of prehypertension (elevated blood pressure or high normal BP) according to different classifications in India during 2015–2021: Evidence from the large national surveys
Source: PLoS One. 2025 Jun 11;20(6):e0325437. doi: 10.1371/journal.pone.0325437 (PMC12157057; doi:10.1371/journal.pone.0325437)
Supplement: Attachment — Submitted filename: Response to reviwers file.docx [file pone.0325437.s007.docx]

**Dear Editor and Reviewers,**

**We would like to extend our sincerest gratitude for the invaluable feedback provided for the manuscript. Your constructive comments and insightful suggestions have been instrumental in improving our manuscript.**

| S.No. | Editor’s Comment | Author’s Response |
| --- | --- | --- |
| **1.** | The results could be reorganised such that prevalence is presented first, before risk factors. | Thank you for your comment.  We want to submit that Prevalence is presented before risk factor only (Figure-1, Table 2 & Table 3 shows prevalence and risk factors respectively) |
| **2.** | As you did not conduct the data collection yourselves, I do not know whether you are able to answer the reviewer's question regarding what was done for respondents with elevated blood pressure. | Thank you this comment.  **Yes we were able to answer this question very well** and the explaination is here;  “It is a secondary analysis of population-based data from India conducted on data sets of NFHS-4 and NFHS-5.  The individuals with hypertension were encouraged to see the doctor for full evaluation as per the NFHS survey.  Please refer to the document (reference no 21, page 46).  No specific intervention for individuals with prehypertension was mentioned in either document.” |
| **3.** | In the methods section (lines 192-193) you state the predictor variables included tobacco and alcohol consumption, yet these variables are missing from the data analyses. Given that you suggest these factors may be the cause for sex differences in hypertension, it would be useful to include these data in your analyses ("Possible reasons for the higher prevalence in men can be higher likelihood of biological and behavioural risk  factors like physical inactivity, smoking habits, and alcohol intake"). | Thank you for this important comment and suggestion.  We didn’t include lifestyle risk factors like alcohol, tobacco and others in our analysis **(with humble submission, *now it will not be feasible as it will disturb whole manuscript.)*** We have done corrections in methods regarding predictor variables.  Mention of these factors has been given in discussion to explain the gender differences!! |
| **4.** | For the logistic regression, please describe which assumptions were checked and whether they were met. | We checked the nature of outcome variable, which is binary (0 and 1) in nature. For example, our outcome variable ‘hypertension’ recoded in either ‘yes’ or ‘no’.  Further, the rationale of using logistic regression, we wanted to see adjusted effect of different predictors on pre-hypertension. |
| **5.** | Please present the overall model significance and overall model fit. | Thank you for this comment, we have added significance of model and model fit in the result section in the revision. |
| **6.** | Please label the significant results more clearly in Table 3. If I understand correctly, you currently have every column labelled as "all the values are significant at 99% CI" with exceptions (95% or non-significant) marked. I think it would be easier to interpret if every significant result was marked with an asterisk or, better yet, report the precise p values. | Thank you for this comment; we have revised the table 3 as per your suggestion. |
| **7.** | As the odds ratios are presented in the table, they do not need repeating in the text, which will make the text of the results section easier to read. | Thank you for kind suggestion.  Correction done |
| **8.** | Please proofread your manuscript carefully. For example, at the beginning of the results section:  "Data from NFHS 4 (2015-2016) and NFHS -5 (were available for 811,591 individuals aged 15 years and above and 1,852,845 individuals respectively."  Note the open parenthesis after NFHS -5  Also please format numbers as 123,456 (not 1,23,456). | Thank you for the comment.  Correction done |

| S.No. | Reviewer’s Comment | Author’s Response |
| --- | --- | --- |
| 1. | Title: Needs resentencing. | Thank you this important comment.  Title has been revised accordingly. |
| 2. | Abstract: Is inconsistent and lacks clarity. | Thank you for highlighting this concern.  Abstract has been revised accordingly for all headings to make it consistent and clear. |
| 3. | Background: Incomplete e.g. contexts are missed. | Thank you for this important concern.  Background has been revised and context is reframed and defined more clearly.  (In addition, to best of our knowledge, no study is available comparing all classification (JNC7, AHA/ACC and IGH-IV) with such large sample size with map diagrams for excellent data visulaization state and distrcit level.) |
| 4. | Methods: Relatively good and Ethics needs justification. What was done for those with elevated blood pressure? | Thank you for appreciation.  **Regarding ethics- Justification is added from in ethics section**. “Both the surveys have received ethical clearance from Institutions Review Board (IRB) of IIPS, Mumbai, India. Also, written informed consent was obtained from all eligible participants before the survey.”  The individuals with hypertension were encouraged to see the doctor for full evaluation as per the NFHS survey.  Please refer to the document (reference no 21, page 46).  No specific intervention for individuals with prehypertension was mentioned in either document.” |
| 5. | Result: Not logical E.g. Factors associated can be presented at the end of the results section. | Thank you for the comment.  Prevalence and Factors associated is presented for all three classifications , hence presented in that order.  Spatial distribution results is presented at end of results as prevelance of Prehypertension was showed in map diagrams using only one classification (JNC 7).  **So to avoid any confusion, we presented factors associated after prevalence as per all classifications.** |
| 6. | Discussion: Needs major revision and further enrichment. | Thank you for your kind suggestion.  Major revision is done.  Discussion is revised for all findings , explanation for each finding is further improved and new references added.  **One important point is added about most recent ESC guidelines in discussion (sep 2024) which further increase weightage in reference of using different classifications in our study.** |
