# Supplementary material for: Prevalence and determinants of prehypertension (elevated blood pressure or high normal BP) according to different classifications in India during 2015–2021: Evidence from the large national surveys
Source: PLoS One. 2025 Jun 11;20(6):e0325437. doi: 10.1371/journal.pone.0325437 (PMC12157057; doi:10.1371/journal.pone.0325437)
Supplement: Attachment — Submitted filename: Response to Reviewers file .docx [file pone.0325437.s008.docx]

**Dear Editor and Reviewers,**

**We would like to extend our sincere gratitude for the invaluable feedback provided for the manuscript. Your constructive comments and insightful suggestions have been instrumental in improving our manuscript.**

| S.No. | Additional Editor’s Comment | Author’s Response |
| --- | --- | --- |
| 1. | Write the result section in the summary form in the abstract. | Thank you for your kind suggestion.  We have revised and written results in the summary form (individual % for states have been deleted to shorten the section).  We also want to humbly state that the results section has highlighted about prevalence in all the states across India (so write-up has increased little). |
| 2. | The NFHS IV and V surveys were conducted using a sample. How does sampling bias affect the prevalence estimates? It may be mentioned in the study's limitations. | Thank you for this important comment and suggestion.  We have included about sampling bias ‘limitations of the study’ in the revision. [ Lines 591-95] |
| 3. | The prevalence estimates lack 95% confidence intervals. In Table 2, mention the unit for prevalence in the column heading. | Thank you for this comment, we have done revision is in table 2. |
| 4. | In Table 3, the age group classifications are 15 to 29, 30-39, 40 to 59, and 50 and above. There is an overlap in the class interval 40 to 59 and 50 and above. Check it. | Thank you for this comment.  We have checked carefully; class intervals are defined as mentioned. |
| 5. | Why were three different classifications of hypertension used for analysis? The odds ratios differ between the various classifications of hypertension in different age groups. According to the JNC 7 and IGH-IV classification for age groups 30-39, 40 to 59, and 60 and above, the odds ratios are >1, and according to the classification of AHA/ACC, they are protective. How can this type of controversial finding be interpreted? | Thank you for this comment.  Different classifications and cut-offs determine the prevalence of prehypertension and, subsequently, policymaking for controlling and preventing hypertension, and formulating screening programs.  Thus, it is critical that uniform and appropriate cut offs be determined for prehypertension.  Strong rationale is given in introduction.  If we see the classifications, the cut off for pre-hypertension as per JNC and IGH IV is more or less same where ACC follows the very different cut off for the classification for the same. As age advances, the risk of pre-hypertension is increasing. Further, as per ACC criteria, may be people are more shifting from pre-hypertension to hypertension that’s why lesser odds found among higher age groups. |
| 6. | Provide high-resolution images to have clear visibility. | Thank you for your important comment.  We have provided high –resolution images. |
|  |  |  |

Reviewer #2: Review Comments

| 1. | Abstract: Lacks clear clarity | Thank you for highlighting this concern.  Abstract has been revised for all headings for clear clarity.  Revision done for background, methods explained further for more clarity; results have been revised and written in summary form for better readability and conclusion is revised as per objectives and findings of our study.  [Lines 31-33, 41-44 and 67-70] |
| --- | --- | --- |
| 2. | Background: Weak for relevance, link and contextual factors | Thank you for this important comment.  Revision is done to provide strong link, relevance and contextual factors meticulously. [Lines 121-127, 133-137]   - Strong and recent problem statement has been given and described in detail. - **We have described in the introduction, the consequences for having different classifications for prehypertension.** Various classifications have been defined with issues around different terminology and cut-off values of BP. - Strong relevance has been given and revised further meticulously to highlight the importance of this topic   [Lines 121-127, 133-137, 140-151  To add, strong rationale for the study has been provided in the introduction and highlighted the fact that new estimates will be of **important public health concern, which can have huge implications on population health outcome and these various cut-off values need to be streamlined**. In addition, to best of our knowledge no study is available comparing all classification with such large sample size.  Moreover, ArcGIS was used to create maps to visually depict the differences when comparing the NFHS-4 to NFHS-5 and GeoDa was also used to compute local indicators of spatial association, which is a **novel way to present data.** |
| 3. | Methods: Not explicit (not fully or clearly revealed or expressed) | Thank you for this comment.  Regarding this comment, we want to submit humbly that in last revision, we were appreciated for methods (**“ methods: relatively good”).**  In methods we have clearly stated in detail –  1. Data source- Data from NFHS-4 and NFHS-5 is used.  2. Study population and procedures- details have been provided and References 21 and 22 cited in the methodology provide detailed information regarding the sampling techniques, study population and sample size for further reading**.**  **(*Also*** *comment from one of the reviewer’-***‘*The authors must be commended for mentioning the study population and procedures in detail****)*  3. Outcome (**S1 table** is added for details of classification and cutoffs) and Predictor variables;  4. Statistical analysis defines fully about analysis of variable and **further revision is done** to express methods **(**supplementary file (S2 text) elaborating the concepts of LISA significance and Moran’s Index has been added to explain in detail about district level prevalence and spatial and cluster maps for further reading)  5.Clear statement of ethics |
| 4. | Results and discussion: Not comprehensive and lacks theoretical as well as practical implications | Thank you for this comment.    We want to submit that results are presented in very detailed manner as follows (reference taken from previous studies published in Plos- One on NFHS analysis and further, we have followed this pattern from paper published from highly reputed journals.)   1. **Sample characteristics** - in written detail for both surveys. 2. **Prevalence of Prehypertension and its trends between NFHS-4 and NFHS-5**- all three classifications (JNC 7, 2017 ACC/AHA and IGH-IV) estimates with respect to independent variables 3. Association of risk factors with prehypertension- with explanation about all risk factors with respect to both surveys and all classifications 4. Spatial distribution of prehypertension trends in India – has been narrated in comprehensive manner (two detailed paragraphs) about prevalence of prehypertension in all states, **Moran’s Index, LISA cluster and significance (which is novel method of representing** data showing ‘hot-spots’ and ‘cold- spots’)  - In note of Table-2 – Details of all three classifications in given to understand different terminology and cutoffs while reading result. - Further revision is done in some sections of result.   **Practical implications of results-**   - Map diagrams give excellent visual evidence about prevalence of prehypertension to guide policymakers to take appropriate tailored actions and implement strategies. - We have presented the district level prevalence of pre-hypertension and ArcGIS used to create maps to visually depict the differences when comparing the NFHS-4 to NFHS-5. - GeoDa used to compute local indicators of spatial association, which is a novel way to present data.   **About discussion we want to kindly submit following points-**   - Further revision is done and one more reference is added [Lines 425-427; 451-455, 468-470, 609-610; reference 83] - All implications have been described in detail objective wise. - The results from our study have highlighted important evidence about high burden of prehypertension and the varied estimates of prehypertension (and related terms) due to different cutoffs of blood pressure used by JNC, ACC/AHA and IGH-IV. - These various classifications and definitions given by JNC 7, 2017 ACC/AHA, 2019 IGH-IV and now ESC 2024 add to the confusion in clinical settings in management of prehypertension and to policy makers for comparing data regionally and globally and need to be streamlined. - Various risk factors have been described, assessment of RFs are required for targeted tailored actions. - Maps diagram provide excellent data visualization of hot spots and cold spots of prehypertension at state and district level. - **Recommendations -** from results are narrated in details about framing uniform cutoffs guidelines, screening programs, and also included the inputs from National initiatives being implemented in India under the National Program- Non Communicable Diseases (NP-NCD). Further, quality research is needed for to decide cost-effectives interventions for to manage prehypertension. - Most recent ESC guidelines (September 2024)- introduced one more definition- not coinciding with any previous guidelines as mentioned in S1 text. This will further add to the further confusion in clinical settings and to policy makers. - **One important point is added about most recent ESC guidelines in discussion (sep 2024)which further increase weightage in reference of using different classifications in our study.** |
