# Supplementary material for: Prevalence and determinants of prehypertension (elevated blood pressure or high normal BP) according to different classifications in India during 2015–2021: Evidence from the large national surveys
Source: PLoS One. 2025 Jun 11;20(6):e0325437. doi: 10.1371/journal.pone.0325437 (PMC12157057; doi:10.1371/journal.pone.0325437)
Supplement: Attachment — Submitted filename: Response to reviwers filee.docx [file pone.0325437.s009.docx]

**Dear Editor and Reviewers,**

**We would like to extend our sincere gratitude for the invaluable feedback provided for the manuscript. Your constructive comments and insightful suggestions have been instrumental in improving our manuscript. Each comment has been answered point wise and in detail.**

| **S.No.** | **Reviewer’s Comment** | **Author’s Response** |
| --- | --- | --- |
| Additional Editor Comments: | |  |
| 1. | It is recommended that the 95% confidence interval (CI) of the prevalence of prehypertension/elevated BP/high normal according to different classifications be mentioned in the results. | Thank you for this comment.  Due to space limitations in table (**Table 2 has 10 columns,** we have compared three different classifications and cutoffs across two surveys, NHFS-4 & 5), confidence intervals were not included alongside prevalence estimates.  Table-3 (association of selected risk factors of prehypertension) has included CIs.  **Its our humble submission, that to make table more readable and presentable, confidence intervals were not included** |
| 2. | Please check the age groups in Tables 1 and 3. After the ‘40 to 59’ age group, '50 or more’ is mentioned. Is it ‘40 to 49’ as mentioned in Table 2? | Thank you for this important comment.  Correction is done and included in the revision.  The age group is ’40-49’ years  We have checked all values again. |
| 3. | Check the total for the age group and educational status; it is not 811591 (NFHS-4 data). In Table 1, the total sample of the ≥50 age group is mentioned as 850,300. Is it a typo error? It is recommended that authors should recheck all values | Thank you for this important comment.  Correction is done and included in the revision(it was a typo error).  We have checked all values again. |
| Reviewer #4: Major Comments | |  |
| 1. | **Introduction:**  - The introduction is overly lengthy and reads more like a review rather than a concise background to the study. The authors should streamline it by focusing on the study’s aim and removing unnecessary details.    -When discussing the different blood pressure cutoffs, introduce a new paragraph to highlight the classification differences, particularly for prehypertension. This will improve readability and logical flow. | Thank you for this kind suggestion.   - Introduction is revised accordingly and streamlined focusing on aim of the study (number of words are reduced and unnecessary details are removed). - We have described the introduction as follows; **global, current country statistics,** strong and recent problem statement has been given and described in detail *(it is written as answer to one of comment in last review*), strong rationale and the **consequences for having different classifications is given.** - Different classifications, cutoffs and terminology (JNC,AHA,ESC,ISH) are explained in detail is **new paragraph**. [Line 104-115]; prehypertension (as used by JNC) is described in beginning of this new paragraph.   **Supplementary table (S1 table)** is added in introduction for details of classification and cutoffs for further reading and clarity. [Line 113] |
| 2. | **Methods:**  - The manuscript does not mention the ethical approval code in the Methods section. Please include the specific ethics approval reference number from the institutional review board. | Thank you for this comment.   - The data source for our analysis was national surveys (NFHS-4 and NFHS-5) conducted under supervision of MoHFW (Ministry of Health and family welfare), the Government of India and managed by the International Institute for Population Sciences (IIPS) in Mumbai, India. - “**Both the surveys have received ethical clearance from Institutions Review Board (IRB) of IIPS, Mumbai, India”.** The anonymized dataset is freely available in the public domain through the Demographic and Health Surveys (DHS) Program (see data availability statement). - **Since we did not acquire any primary data, ethical approval for our study is not mandatory.**   **Clear statement of ethics mentioned in methods [Line 248-256]** |
|  | -The section on blood pressure measurement needs further elaboration. Clarify the methodology used to measure BP, including the device specifications (already mentioned) and standard procedures followed to minimize measurement errors (e.g., rest periods, multiple readings). | Thank you for this important comment  **Details about device specifications, rest periods; multiple readings are all mentioned in methods.**   - The process and instrument for measuring blood pressure were the same for NFHS 4 and 5. BP reading was measured for women and men above 15 years of age in the selected households using a standard digital blood pressure device. [176-179] - **BP was measured three times for each individual (using Omron™ HEM-8712), with at least five minutes gap between each measurement. [Line 179-181]** - The average blood pressure computed for the second and third readings has been considered. Studies have reported that the first reading of BP is usually high due to anxiety. [Line 187-189]   All the details regarding training, measurements, and procedures followed are described in detail in the NFHS 4 and 5 documents.(**Reference no. 21 and 22 for further reading.)** |
|  | - Provide more details on how cases of early-stage secondary hypertension were differentiated from prehypertension, as this could affect prevalence estimates. | Thank you for this comment   - We want to submit that, the present study aimed to **estimate the prevalence of prehypertension** (elevated blood pressure or high normal BP) and **its determinants** based on different standard classifications using the large population-based data from the fourth and fifth rounds of National Family Health Surveys (NHFS), India. - We also intended to present spatial and cluster maps to identify the trends of prehypertension between NFHS-4 and NFHS-5 for India as well as at the state and district levels to guide policymakers.   **Early-stage secondary hypertension is not an objective of this analysis.** (There is a presence of a ‘specific underlying cause’ in secondary hypertension, which is beyond the scope of this analysis) |
| 3. | **Results:** - The findings should be age-standardized to ensure they are representative of the entire Indian population. This will improve generalizability and comparability with other studies. | Thank you for this comment  The primary goal of the study is to describe the actual age-specific burden or prevalence in the population.  Therefore, reporting crude (unstandardized) rates is more appropriate and informative for policy or programmatic decision-making. |
|  | - The results should be discussed in relation to demographic transitions in India. How does the aging population and urbanization influence prehypertension trends? | Thank you for this important comment  The demographic transition and aging population will increase burden of non-communicable diseases which is well discussed in context to our findings.   - The high burden of prehypertension and subsequently hypertension is going to be major driver for cardiovascular diseases epidemic in country. [ Lines 459-460] - The comparison of this data between developed and developing parts of the world indicates that the burden of Non-Communicable Diseases is alarmingly increasing in developing countries as well. **Developing countries like India already suffering from communicable diseases, NCDs will impose huge disease and financial burden on the patients and healthcare systems**.[ Lines 479-483] - Influence of urbanization on prehypertension trends has been discussed in details for prevalence of prehypertension for various determinants, across different social groups socio-economic strata groups and across different states. [Lines 546-557] |
| 4. | **Discussion and Comparisons with Other Studies**: - The discussion would benefit from comparisons with other high-population countries in the region, such as Iran and China. How do the findings align with or differ from their prevalence trends? | Thank you for this comment on comparison of results with other high-population countries.   - Authors have well discussed the results with other countries including high-population countries. [Line 466-483] - Estimates for prevalence of prehypertension lie in the range of 33-47% from other South Asian countries (China, Taiwan, Vietnam, Korea, Iran, Bangladesh) and adjacent low-middle income countries between 2005- 2015. [467-469]   Our estimates align almost with high population country like china. |
|  | Expand on the potential policy implications of these findings. How can the variations in prehypertension definitions impact clinical guidelines and public health strategies? | Thank you for this important comment.   - **Different classifications** and cut-offs determine the prevalence of prehypertension and, subsequently, policymaking for controlling and preventing hypertension, and formulating screening programs. [Lines 126-130, 557-559; 597-599] - **Strong rationale** for the study has been provided in the introduction and highlighted the fact that new estimates will be of **important public health concern, which can have huge implications on population health outcome and these various cut-off values need to be streamlined**. In addition, to best of our knowledge no study is available comparing all classification with such large sample size.[139-142] - **Map diagrams** give excellent visual evidence about prevalence of prehypertension to guide policymakers to take appropriate tailored actions and implement strategies. - These findings also highlighted important evidence about the varied estimates of prehypertension (and related terms) due to different cutoffs of blood pressure used by JNC, ACC/AHA and IGH-IV.[Lines 420-422] - The increase in these burden estimates alarms the concern for identifying individuals with prehypertension, to raise awareness and taking swift measures to halt the progression to hypertension to reduce premature morbidity and mortality.[lines 463-465] - **As well explained in discussion;** these various classifications and definitions given by JNC 7, 2017 ACC/AHA, 2019 IGH-IV and now ESC 2024 impact clinical guidelines and have huge implications on policy issues and need to be streamlined. [**Lines 423-450]** - **One important point is added about most recent ESC guidelines in discussion (sep 2024) which further increase weightage in reference of using different classifications in our study. [Lines 442-446]** - Physicians in clinical practice and primary health care setting should routinely measure blood pressure; identify patients with prehypertension and strong advocacy of lifestyle modifications is required. The National Health Policy of India, 2017, aimed to reduce premature deaths due to cardiovascular causes by 25% and treat ≥ 80% of patients with hypertension by 2025. Also, insightful data from our analysis of regional heterogeneity across the country will guide targeted approaches and cost-effective interventions for control of the epidemic of hypertension. [624-630] |
| **5.** | **Minor Comments** - Ensure consistency in terminology (prehypertension, elevated BP, high normal BP). Define these terms clearly at the beginning of the study. | Thank you for this comment    **THE AIM OF THIS UNIQUE ANALYSIS IS HIGHLIGHT THESE TERMS (**PREHYPERTENSION, ELEVATED BP, HIGH NORMAL BP)**, THE CONFUSION AND THE CONSEQUENCES.**   - Different classifications (JNC,AHA,ESC,ISH), cutoffs and terminology (Prehypertension, elevated BP, high normal BP) are explained in detail is **new paragraph**. [Line 104-115]; prehypertension (as used by JNC) is described in beginning of this new paragraph. - **Supplementary table (S1 table)** is added in introduction for details of classification and cutoffs for further reading and clarity. [Line 113] |
|  | Improve the clarity of some statistical explanations, particularly in the spatial analysis section.    -There are a few minor typographical errors (e.g., "o,it" should be "omit", "Metntion" should be "Mention", "secion" should be "section"). A thorough proofread will enhance readability. | Thank you for this comment  In statistical analysis section, **we have added supplementary document (S2) elaborating the concepts of LISA significance and Moran’s Index.**  GIS is used to visualize and analyze spatial patterns in the data, enabling map-based representation.  To display the district-level prevalence of prehypertension, ArcGIS was used to create a map to see the changes from NFHS-4 to NFHS-5 GeoDa software (spatial and cluster maps) version 1.20.0.20 was used to compute Local Indicators of Spatial Association (LISA). [Lines 236-239]  We obtained a LISA significance cluster to identify the clusters of locations with a higher prevalence of prehypertension. The significant value of LISA helps to identify the set of contiguous location, which are labeled as “local spatial clusters” or “Hot spots”. We also obtained Moran's index to explain the data's overall clustering and project the strength and patterns of spatial autocorrelation. Positive and significant Moran’s I value depicts clustering of like values and these values can be either high or low or even combination of both. [ Lines 239-245]  Typographical errors are corrected and thorough proof reading is done |
